# Supplementary material for: Hot-Melt Extrusion Process Fluctuations and Their Impact on Critical Quality Attributes of Filaments and 3D-Printed Dosage Forms
Source: Pharmaceutics. 2020 Jun 3;12(6):511. doi: 10.3390/pharmaceutics12060511 (PMC7357138; doi:10.3390/pharmaceutics12060511)
Supplement: Supplementary file 1 [file pharmaceutics-12-00511-s001.pdf]

pharmaceutics

Supplementary Material: Hot-Melt Extrusion Process Fluctuations and their Impact on Critical Quality Attributes of Filaments and 3D-printed Dosage Forms

Hanna Ponsar, Raphael Wiedey and Julian Quodbach *

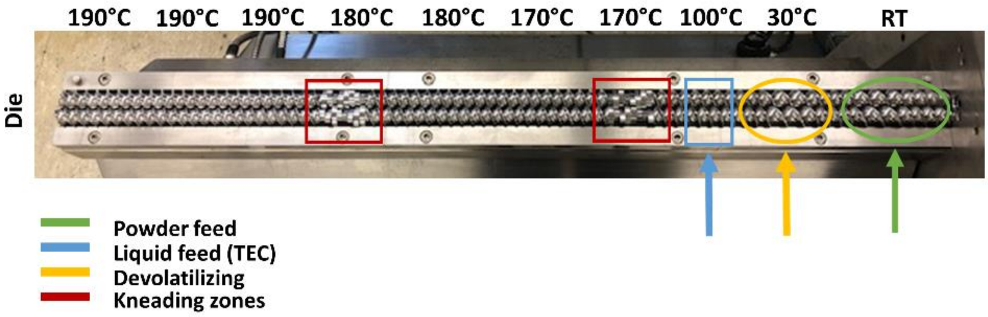

Figure S1. Screw- and temperature configuration and barrel sections (RT = room temperature).
